# Through-and-Through Brachio/Radio-Femoral Access Technique for Endovascular Recanalization of the Left Subclavian Artery

**DOI:** 10.1055/s-0042-1743198

**Published:** 2022-05-31

**Authors:** Young Erben, Michelle Lin, Camila Franco-Mesa, Josephine F. Huang, Sukhwinder S. Sandhu, David Miller, Rabih G. Tawk

**Affiliations:** 1 Division of Vascular and Endovascular Surgery, Mayo Clinic, Jacksonville, Florida; 2 Department of Neurology, Mayo Clinic, Jacksonville, Florida; 3 Department of Radiology, Mayo Clinic, Jacksonville, Florida; 4 Department of Neurosurgery, Mayo Clinic, Jacksonville, Florida

**Keywords:** endovascular, through-and-through access, left subclavian artery recanalization

## Abstract

The authors illustrate the use of through-and-through brachio/radio-femoral access technique in two patients who presented with subclavian steal syndrome. This is an additional tool in the armamentarium of the endovascular specialist to improve management of complex cases with subclavian steal syndrome. This technique provides several advantages to improve efficiency and precision of the procedure while reserving the open surgical bypass option if needed.

## Introduction

Through-and-through wire technique for endovascular interventions is commonly used for endovascular aortic and peripheral interventions. 1 2 The purpose of this report was to enhance this literature and suggest an alternative pathway to recanalization of an occluded vessel/stent and precise angioplasty and/or placement of a stent.

## Case Presentations

### Case 1

**Fig. 1:**
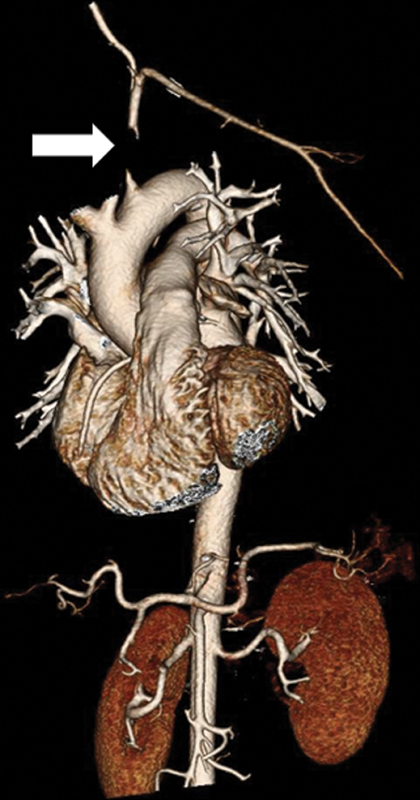
Computed tomography angiography three-dimensional reconstruction of the aorta and the left subclavian artery occlusion (white arrow).

**Fig. 2:**
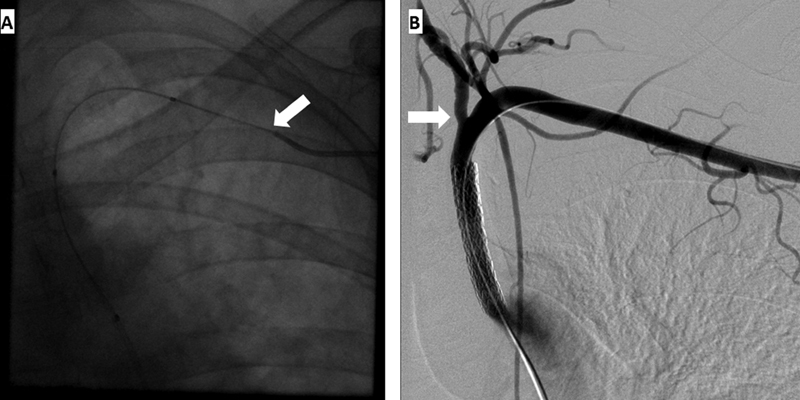
( **A** ) Accessing of the glidewire into the lumen of the Kumpe catheter through the left brachial artery (white arrow) coming from the left common femoral artery and ( **B** ) angiogram of the already deployed 6-mm iCast covered stent in the left subclavian artery (white arrow depicts the left vertebral artery).

A 67-year-old female patient with medically managed dyslipidemia and hypertension presented for evaluation for new left upper extremity tiredness, heaviness, and pain with activity. Cross-sectional imaging in the form of computed tomography angiography (CTA) and three-dimensional reconstruction demonstrated an occlusion of the left subclavian artery (LSCA) ( Fig. 1 ). Recanalization of the LSCA was initiated through a left brachial artery approach. However, there was inability to assure whether the soft-angled glidewire was traversing the true lumen or a subintimal plane of this vessel. Therefore, access was gained through the left common femoral artery (LCFA), and in a retrograde fashion through the aorta, the stump of the LSCA was probed with a second glidewire and recanalization of the LSCA was facilitated. Once access through the true lumen of the LSCA was achieved, the glidewire from the LCFA was used to probe the tip of the Kumpe catheter from the left brachial artery, and by traversing this catheter, we obtained through-and-through access from the left brachial to the LCFA ( Fig. 2A ). With this through-and-through wire in place, precise deployment of a 6-mm covered iCast (Atrium Medical Corporation, Merrimack, NH) stent ( Fig. 2B ) was facilitated without risking coverage of the left vertebral artery by gently tugging from each end at the through-and-through wire.

### Case 2

**Fig. 3:**
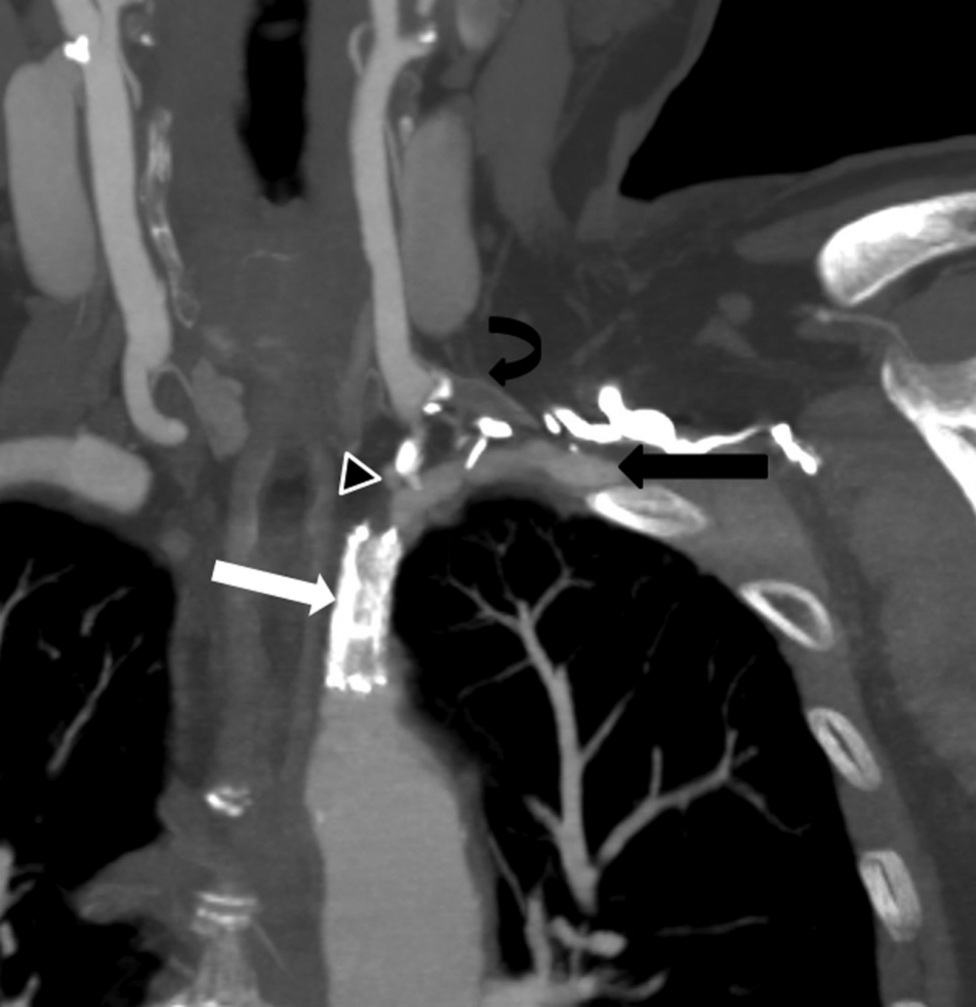
Curved reformat computed tomography angiography with maximal intensity projections at 8 mm demonstrates occluded left subclavian artery stent (white arrow) and occluded left common carotid artery to subclavian artery bypass graft (curved black arrow). The left subclavian artery (black arrow) is predominately supplied by retrograde flow as seen at the left vertebral artery origin (black arrowhead).

**Fig. 4:**
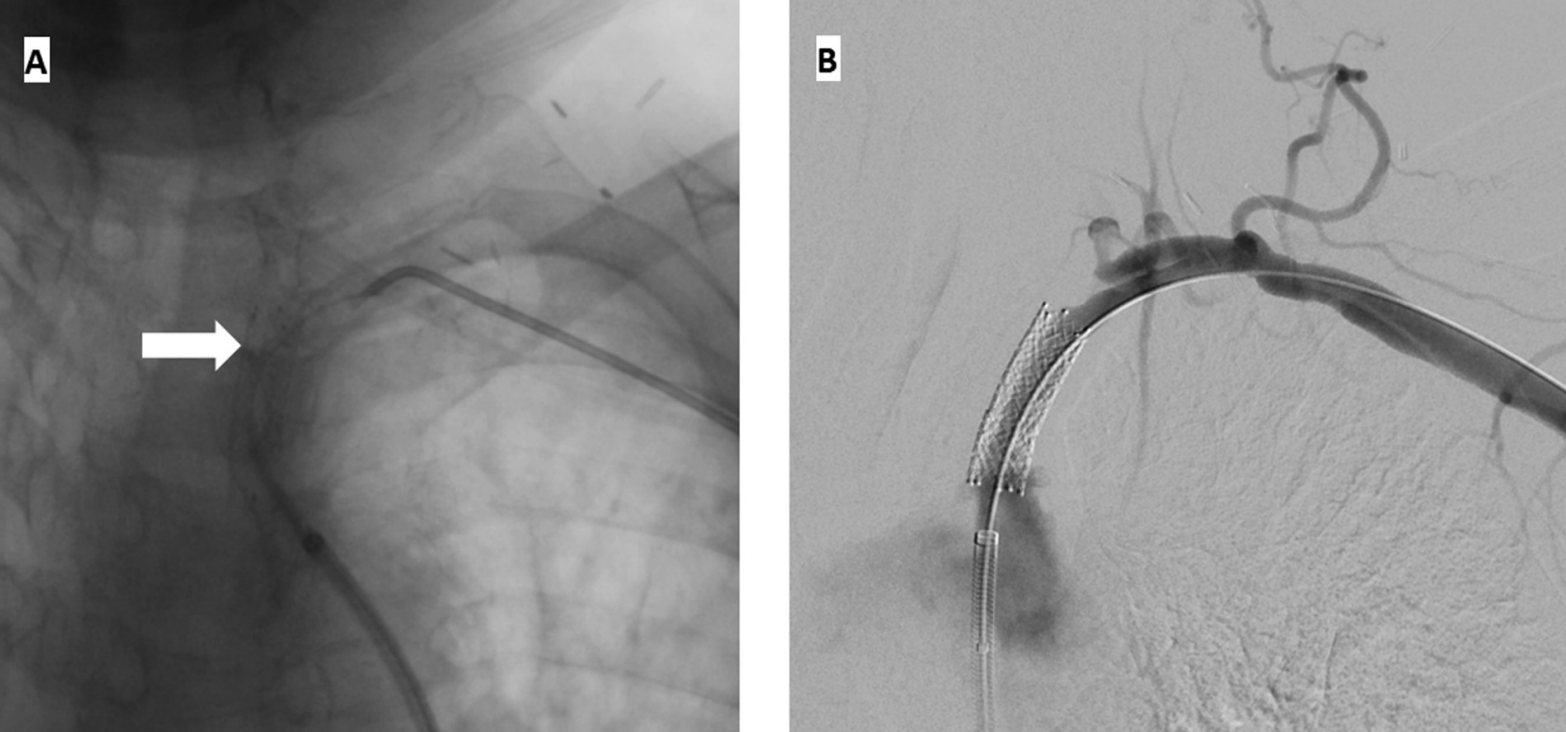
( **A** ) The stiff end of the glidewire (white arrow) was used to cross the occluded left subclavian artery stent with the support of a stiff guide catheter from the left common femoral artery approach. ( **B** ) Once through-and-through access from the left radial artery to the left common femoral artery was obtained, the stent in the left subclavian artery was angioplastied.

A 72-year-old female patient with a previously failed LSCA stent and left carotid to subclavian artery bypass presented for evaluation for new left upper extremity tiredness and a syncopal episode. CTA demonstrated an occlusion of the LSCA stent and left carotid to subclavian artery bypass ( Fig. 3 ). Recanalization of the LSCA stent was performed through a left radial artery and LCFA approach. In this instance, a stiff guide catheter was placed right at the origin of the LSCA stent and using the back end of the glidewire access was obtained into the LSCA ( Fig. 4A ). Again, through-and-through access from the left radial to the LCFA was obtained and the LSCA stent was angioplastied ( Fig. 4B ).

## Discussion

Ischemic stroke is one of the leading causes of death and long-term disability worldwide. 3 Carotid stenosis is responsible for a 2% risk of stroke per year. We present this case of rapid progression of disease in a patient who would not, under the current guidelines, have been subject to routine carotid screening. 1 Furthermore, it presents with intraplaque hemorrhage, a marker for high risk of stroke and plaque progression. 4 This case illustrates the value of additional imaging studies in patients who demonstrate atherosclerosis detected by screening ultrasound. CTA is widely available, relatively inexpensive, and specific for detection of cervical internal carotid artery stenosis. Magnetic resonance angiography (MRA) with vessel wall imaging is increasingly utilized to better characterize carotid plaques to identify vulnerable lesions especially with borderline luminal stenosis. MRA is the imaging modality of choice to best detect intraplaque hemorrhage, 5 which is known to correlate with subsequent ischemic cerebrovascular events. 4
